# Longitudinal changes in sarcopenia was associated with survival among cirrhotic patients

**DOI:** 10.3389/fnut.2024.1375994

**Published:** 2024-05-30

**Authors:** Minjie Jiang, Xin Hua, Muchen Wu, Jing Wu, Xiaotong Xu, Juan Li, Qinghua Meng

**Affiliations:** ^1^Department of Medical Oncology, Beijing Youan Hospital, Capital Medical University, Beijing, China; ^2^Beijing Institute of Hepatology, Beijing, China; ^3^Department of Clinical Nutrition, Beijing Youan Hospital, Capital Medical University, Beijing, China; ^4^Department of Hepatology, Beijing Youan Hospital, Capital Medical University, Beijing, China

**Keywords:** sarcopenia, liver cirrhosis, longitudinal analysis, clinical outcomes, mortality

## Abstract

**Background:**

Sarcopenia is common in patients with liver cirrhosis and is an independent predictor of multiple clinical outcomes. Most studies to date have used a static assessment of sarcopenia. However, there is very limited data evaluating the temporal course of muscle area in cirrhosis. To bridge this gap in clinical studies, we performed a longitudinal analysis to evaluate the impact of changes in sarcopenia for cirrhotic patients.

**Methods:**

Adult patients with clinically diagnosed liver cirrhosis who underwent at least 2 abdominal computed tomography (CT) scans in the hospital were enrolled. The interval between the two abdominal scans was 6 ± 1 months. Patients were categorized into persistent non-sarcopenia, new-onset sarcopenia, sarcopenia to non-sarcopenia, and persistent sarcopenia based on changes in sarcopenia. Kaplan–Meier method and Log-rank tests were used to separately compare unadjusted survival curves by different statuses of sarcopenia. Cox regression analysis was performed to assess the associations between different states of sarcopenia and overall mortality. The association between persistent non-sarcopenia and new-onset sarcopenia was analyzed by multivariate logistic regression analysis.

**Results:**

A total of 307 patients were included for analysis. At the second assessment, 10.10% (31/307) patients were new-onset sarcopenia, 27.69% (85/307) with persistent sarcopenia status, while 13.03% (40/307) patients with sarcopenia developed non-sarcopenia and 49.19% (151/307) with persistent non-sarcopenia status. The overall survival rate was significantly lower in the persistent sarcopenia and new-onset sarcopenia than in the non-sarcopenia group and sarcopenia to non-sarcopenia group (*p* < 0.001). Persistent sarcopenia (HR 5.799, 95%CI 1.563–21.521, *p* = 0.009) and new onset sarcopenia (HR 5.205, 95%CI 1.482–18.282, *p* = 0.010) were identified as poor prognostic factors for cirrhotic patients. The etiology of cirrhosis and the initial skeletal muscle mass were independent risk factors for new-onset sarcopenia.

**Conclusion:**

Sarcopenia is a dynamically changing process in patients with cirrhosis. Persistent and new-onset sarcopenia were independently and robustly associated with overall survival.

## Introduction

Sarcopenia was initially described in 1989 as the “losses of skeletal muscle mass with aging” (1). The definition has since evolved, and the European Working Group on Sarcopenia defined sarcopenia as “a progressive and generalized skeletal muscle disorder associated with an increased likelihood of adverse outcomes” combining both muscle mass and muscular function (2, 3). In 2016, sarcopenia has been registered as an independent disease by the new international statistical classification of diseases and related health problems, 10th revision (ICD-10-CM) (4, 5). Sarcopenia is a relatively common condition and is associated with increased adverse outcomes including falls, functional decline, frailty, and mortality, and imposes an enormous public health burden (6).

The age-related reduction of skeletal muscle mass is defined as primary sarcopenia, whereas muscle atrophy related to activity, chronic illness, malignant tumor, and nutrition status are classified as secondary sarcopenia (7, 8). Sarcopenia that develops from liver cirrhosis is an example of secondary sarcopenia (8). Sarcopenia in patients with liver cirrhosis is multifactorial, and malnutrition is not the only factor that contributes to sarcopenia (8). Therapies focused on nutritional supplementation have been frequently inadequate in improving survival in cirrhotic patients with sarcopenia (9). The liver plays a critical role in energy metabolism, and its functional integrity is essential for the supply and inter-organ trafficking of macronutrients and their metabolism (10, 11). Cirrhosis is in a state of accelerated starvation, with hepatic glycogen depletion, impaired non-oxidative glucose metabolism, decreased protein synthesis, and increased gluconeogenesis from amino acids, which contributes to an imbalance of muscle synthesis and breakdown (12–14). Hepatocellular dysfunction and portosystemic shunting also result in biochemical and hormonal perturbations in patients with cirrhosis that contribute to sarcopenia (15, 16). Other factors in cirrhosis such as systemic inflammation, physical inactivity, and environmental/organizational factors can contribute to sarcopenia within or independent of the malnutrition pathway (17). Liver cirrhosis is a major susceptibility condition for the occurrence of secondary sarcopenia (17–19).

The prevalence of sarcopenia in patients with cirrhosis varies widely, with a range of 30% ~ 70%, depending on the etiology of the liver disease, the severity of the liver disease, and the diagnostic criteria utilized (17). Regardless of how sarcopenia is defined, it is associated with a wide spectrum of outcomes in cirrhosis, including poor quality of life, mortality in patients on the liver transplantation waitlist, longer stays in hospital or intensive care unit, increased incidence of infection following liver transplantation, and higher overall healthcare cost (4, 19–22). Over the past few years, sarcopenia has become a topic of prolific exploration in patients with cirrhosis (4). To date, most studies have used statically measured sarcopenia, but recent studies suggest that sarcopenia is a dynamically changing process (23). Therefore, it is critical to assess the clinical meaning of longitudinal changes in sarcopenia, and whether they have prognostic value independent of other measurements. To address this gap in clinical research, we performed a longitudinal study to evaluate the effect of changes in sarcopenia for cirrhotic patients.

## Methods

### Study population

This study was designed as retrospective research that included cirrhotic patients hospitalized between January 1, 2018, and February 28, 2022, at Beijing You-An Hospital, Capital Medical University. Adult patients with clinically diagnosed liver cirrhosis who underwent at least 2 abdominal CT scans in the hospital were enrolled. The interval between the two abdominal scans was 6 ± 1 months. Liver cirrhosis was diagnosed by typical clinical manifestations, laboratory tests, and imaging characteristics. The exclusion criteria included the following: (1) diagnosed with any malignant tumor; (2) long-term bedridden; (3) diagnosed with Acquired Immune Deficiency Syndrome; (4) diagnosed with diabetes; (5) pregnancy or lactation; and (6) any disease that can cause intestinal nutrient absorption disorders. The research was approved by the ethics committee of Beijing You-An Hospital, Capital Medical University (No. LL-2020-079K). The informed consent was waived due to a retrospective study.

### Anthropometric measurements

Height and weight were measured and recorded by nutritionists using standard measurement methods as described by the previous study to determine height (24). In this research, the dry body weight of patients with fluid retention was corrected according to the clinical severity of ascites subtracted a percentage of body weight (reducing 5, 10%, or 15% of the current body weight if mild, moderate, or severe ascites, respectively), and an additional 5% in body weight will be discounted if patients have water retention in the lower limbs (14). The body mass index (BMI) was calculated as the dry weight in kilograms divided by the height in meters squared (kg/m^2^) (25).

### Assessment of skeletal muscle mass

All patients underwent abdominal CT image scanning using a CT scanner (Light-Speed VCT CT 64 Scanner) and were analyzed for a range of body composition metrics using computer-stored CT images. Skeletal muscle was identified through Hounsfield unit thresholds (−29 HU to +150 HU) as the previous study described (19). The skeletal muscle mass at the third lumbar vertebra was calculated by Syngo.via Siemens AG software. The skeletal muscle index at the third lumbar skeletal muscle index (L3-SMI) was calculated as the muscle area at the third lumbar vertebrae (cm^2^) divided by the height in meters squared (m^2^), which has been recommended for the assessment of sarcopenia (19, 22, 26).

### Sarcopenia and longitudinal changes in sarcopenia

The diagnostic criteria of sarcopenia were based on Japan Society of Hepatology guidelines for sarcopenia in liver disease and the optimal cut-off values for L3-SMI were < 42 cm^2^/m^2^ for males and < 38 cm^2^/m^2^ for females (7). The interval between the initial and second skeletal muscle assessments was 6 ± 1 months. According to the changes in skeletal muscle mass, patients were stratified into four groups: (a) persistent non-sarcopenia: non-sarcopenia both in initial and second assessment; (b) new-onset sarcopenia: non-sarcopenia in initial but developed to sarcopenia in second assessment; (c) sarcopenia to non-sarcopenia: sarcopenia in initial and improved to non-sarcopenia in second assessment; and (d) persistent sarcopenia: sarcopenia both in initial and second assessment.

### Data collection

Demographic information, complications, and laboratory results were collected from the medical records. The laboratory data included aspartate amino transferase (AST), alanine aminotransferase (ALT), total bilirubin (TBIL), serum albumin (ALB), serum prealbumin (PA), serum creatinine (Cr), the neutrophil-to-lymphocyte ratio (NLR), and serum hemoglobin (HB). Model End-Stage Liver Disease (MELD) score and Child-Pugh score were calculated using the data recorded in the medical records system and calculated by professional physicians as previously reported (27, 28). A specialized nutrition team to conduct a nutritional assessment and record the results in the medical record. All patients were followed up until May 1, 2023, or until death (for any reason). In our study, all patients who underwent liver transplantation were grouped with the patients that had died. Overall survival time (OS) was measured from the time of the second assessment of sarcopenia until death or the date of administrative censoring.

### Statistical analysis

Mean ± standard deviation was used to express the measurement data if they were normally distributed, and an independent sample Student’s *t*-test was performed for the comparison between the two groups. The median (interquartile range) was used to express the measurement data if they were not normally distributed, and the Mann–Whitney nonparametric test was conducted for the comparison between the two groups. The counting data were analyzed by a Chi-square test. We estimated cumulative incidence of survival rate with the Kaplan–Meier method. Log-rank test was used to separately compare unadjusted survival curves by sarcopenia. Cox regression analysis was also used to assess the associations between sarcopenia and overall mortality, and the results are shown as hazard ratios (HR) with 95% confidence interval (95% CI). The variables with statistical differences after univariate analysis were included in multivariate analysis. Multivariate logistic regression analysis was used to analyze the association between persistent non-sarcopenia and new-onset sarcopenia. Statistical analysis was performed by SPSS Statistics 24 (IBM Corp, Armonk, NY), and *p* < 0.05 was considered statistically significant.

## Results

### Patient characteristics

**Figure 1:**
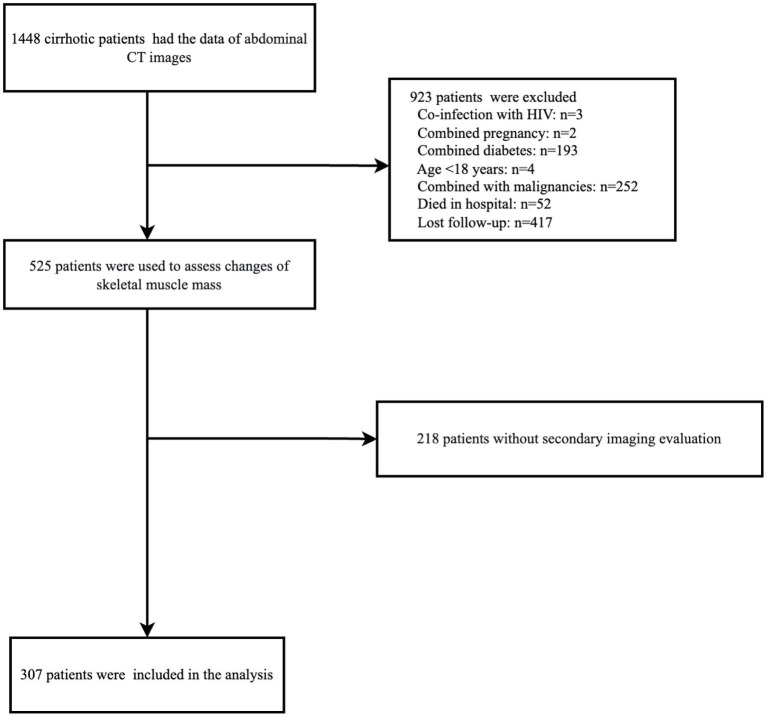
Flow chat of the study.

The 307 patients who met the inclusion criteria and those who did not meet the exclusion criteria and completed all follow-up assessments were included in the analysis. The detailed flow of selection is shown in Figure 1. The demographic, nutritional, and clinical characteristics of all participants are shown in Table 1. Among the included patients, 61.2% (188/307) were male and 38.8% (119/307) were female. The mean age was 57.35 ± 11.81 years and the mean BMI was 22.09 ± 4.74 kg/m^2^. In the current study, the mean L3-SMI was 43.17 ± 8.86 cm^2^/m^2^. The MELD score of the patients included in this study was 15.57 ± 6.1, and the Child score was 8.69 ± 2.03. The most common etiology of cirrhosis was viral liver disease (34.9%), followed by alcoholic liver disease (28.7%) and primary biliary cholangitis (PBC) (13.7%). At baseline, 61.2% (188/307) cirrhotic patients had ascites, 39.7% (122/307) had spontaneous peritonitis (SBP), and 20.8% (64/307) had Hepatic Encephalopathy (HE). In total, 27.4% (84/307) of cirrhotic patients died during the follow-up, and the mean overall survival time was 30.18 ± 14.96 months.

**Table 1:** Clinical characteristics of cirrhotic patients according to change in sarcopenia status.

	Overall patients *N* = 307	Persistent non-sarcopenia *N* = 150	Sarcopenia to non-sarcopenia *N* = 40	Persistent sarcopenia *N* = 86	New-onset sarcopenia *N* = 31	*p*-value
Gender male, *n* (%)	188 (61.2)	102 (68.0)	27 (67.5)	41 (47.7)	18 (58.1)	0.016
Age, years	57.35 ± 11.81	56.63 ± 12.03	55.23 ± 10.93	58.64 ± 11.70	59.94 ± 11.85	0.226
BMI, kg/m^2^ (initial)	22.09 ± 4.74	24.22 ± 4.82	20.93 ± 2.92	19.25 ± 3.68	21.09 ± 3.97	<0.001
BMI, kg/m^2^ (second)	21.75 ± 4.58	23.89 ± 4.42	21.21 ± 2.84	18.80 ± 3.82	18.84 ± 3.83	<0.001
∆ BMI	−0.33 ± 1.24	−0.33 ± 1.21	0.28 ± 6.76	−0.45 ± 1.46	−0.83 ± 0.95	<0.001
L3-SMI, cm^2^/m^2^ (initial)	43.17 ± 8.86	49.28 ± 7.02	38.00 ± 3.25*	34.40 ± 5.15	44.20 ± 4.81*	<0.001
L3-SMI, cm^2^/m^2^ (second)	43.12 ± 9.21	49.30 ± 6.95	45.17 ± 4.50*	33.32 ± 5.40	37.27 ± 3.42*	<0.001
∆ L3-SMI	−0.05 ± 6.18	0.03 ± 5.49	7.17 ± 5.03	−1.08 ± 4.42	−6.93 ± 5.40	<0.001
MELD score (initial)	15.57 ± 6.10	15.59 ± 5.95*	16.68 ± 5.98*	14.94 ± 6.27	15.65 ± 6.51	0.527
MELD score (second)	13.93 ± 6.17	13.64 ± 5.52*	13.99 ± 5.82*	14.52 ± 7.09	13.64 ± 7.02	0.761
∆ MELD score	−1.63 ± 5.78	−1.95 ± 5.60	−2.69 ± 7.00	−0.43 ± 4.82	−2.01 ± 7.01	0.128
Child-Pugh score (initial)	8.69 ± 2.03	8.69 ± 2.02*	9.05 ± 2.12*	8.51 ± 2.04	8.77 ± 1.96	0.580
Child-Pugh score (second)	8.04 ± 2.03	7.76 ± 2.20*	7.73 ± 2.16*	8.60 ± 2.46	8.42 ± 2.29	0.022
∆ Child-Pugh Score	−0.65 ± 2.23	−0.96 ± 2.15	−1.33 ± 2.37	0.093 ± 1.99	−0.35 ± 2.59	0.001
Clinical outcomes						
HE, *n* (%) (initial)	64 (20.8)	29 (19.3)	11 (27.5)	13 (15.1)*	11 (35.5)	0.071
HE, *n* (%) (second)	98 (31.9)	36 (24.0)	10 (25.0)	37 (43.0)*	15 (48.4)	0.003
Ascites, *n* (%) (initial)	188 (61.2)	81 (54.0)	24 (60.0)	65 (75.6)	18 (58.1)	0.012
Ascites, *n* (%) (second)	179 (58.3)	80 (53.3)	17 (42.5)	60 (69.8)	22 (71.0)	0.006
SBP, *n* (%) (initial)	122 (39.7)	61 (41.1)*	15 (37.5)*	32 (37.6)	13 (41.9)	0.937
SBP, *n* (%) (second)	84 (27.4)	35 (23.3)*	5 (12.5)*	32 (37.2)	12 (38.7)	0.008
Death, *n* (%)	84 (27.4)	23 (15.3)	6 (15.0)	43 (50.0)	12 (38.7)	<0.001
OS, months	30.18 ± 14.96	33.19 ± 15.04	34.64 ± 15.18	24.11 ± 12.35	26.92 ± 15.46	<0.001
Serological examination						
AST, U/L (initial)	35.00 (25.00, 61.80)	36.00 (26.00, 67.00)	36.50 (26.50, 79.50)	31.00 (24.00, 45.00)	44.00 (27.00, 71.00)	0.039
AST, U/L (second)	35.00 (26.00, 54.00)	35.50 (25.75, 53.00)	38.00 (26.25, 59.75)	33.50 (27.00, 55.00)	34.00 (25.00, 55.00)	0.888
ALT, U/L (initial)	20.00 (14.00, 35.00)	23.00 (15.00, 40.00)	22.00 (16.25, 38.75)	16.0.00 (12.00, 26.50)	19.00 (13.00, 36.50)	0.001
ALT, U/L (second)	21.00 (14.00, 32.00)	24.00 (15.00, 33.00)	22.50 (16.00, 32.75)	19.50 (13.00, 25.50)	20.00 (13.00, 33.00)	0.225
TBIL, μmol/L (initial)	42.90 (23.60, 84.30)	43.20 (25.40, 84.30)	48.15 (31.08, 100.33)	32.60 (19.75, 79.70)	46.90 (19.90, 140.40)	0.142
TBIL, μmol/L (second)	35.20 (20.80, 66.60)	35.40 (22.58, 63.90)	42.10 (20.53, 78.93)	29.30 (18.55, 74.80)	31.50 (19.10, 66.00)	0.683
ALB, g/L (initial)	30.70 (27.90, 34.40)	30.40 (27.70, 33.80)*	31.00 (27.90, 35.85)*	31.00 (27.75, 34.65)	31.300 (28.70, 33.50)	0.628
ALB, g/L (second)	33.15 (28.80, 37.30)	33.80 (29.70, 37.95)*	34.10 (29.65, 39.93)*	32.35 (27.23, 35.03)	32.10 (29.10, 36.70)	0.011
PA, mg/L (initial)	76.00 (58.00, 109.25)	76.00 (60.00, 115.00)*	81.00 (54.00, 99.00)	83.00 (59.00, 114.00)	63.00 (52.00, 75.00)	0.085
PA, mg/L (second)	88.00 (63.00, 129.00)	93.00 (65.00, 146.00)*	81.50 (65.25, 126.00)	84.00 (57.25, 109.75)	93.00 (55.50, 116.25)	0.444
Cr, μmol/L (initial)	60.00 (49.00, 74.00)	62.00 (52.00, 74.00)	54.50 (45.25, 67.75)	61.00 (50.00, 78.50)	51.00 (46.00, 64.00)	0.045
Cr, μmol/L (second)	61.00 (51.75, 79.25)	62.00 (53.50, 76.00)	57.50 (51.00, 76.75)	66.00 (51.75, 91.00)	57.00 (44.00, 71.00)	0.136
NLR (initial)	2.49 (1.59, 4.29)	2.47 (1.58, 3.71)	2.38 (1.36, 5.10)	2.64 (1.65, 5.20)	2.82 (1.36, 4.02)	0.739
NLR (second)	2.68 (1.72, 4.81)	2.32 (1.70, 3.70)	2.78 (1.62, 6.16)	3.40 (1.77, 7.07)	2.40 (1.59, 6.49)	0.118
HB, g/L (initial)	91.00 (72.00, 113.00)	101.00 (79.00,120.00)*	90.50 (72.00, 113.50)	84.00 (69.00, 106.00)	88.00 (71.00, 109.00)	0.001
HB, g/L (second)	101.00 (78.00, 122.00)	111.00 (83.50, 129.50)*	97.00 (86.25, 121.25)	89.00 (73.00, 109.25)	94.00 (78.00, 121.00)	<0.001

* *p* < 0.05, initial assessment vs second assessment. MELD, model for end-stage liver disease; BMI, body mass index; L3-SMI, the third lumbar skeletal muscle index; AST, aspartate amino transferase; ALT, alanine aminotransferase; TBIL, total bilirubin; ALB, albumin; PA, prealbumin; Cr, creatinine; NLR, the neutrophil-to-lymphocyte ratio; HB, hemoglobin; SBP, spontaneous peritonitis; HE, Hepatic Encephalopathy; OS, overall survival.

### Prevalence and impact of sarcopenia in cirrhosis

Based on the Japan Society of Hepatology guidelines for sarcopenia in liver disease, 41.04% (126/307) patients were diagnosed with sarcopenia at the initial scan. Clinical characteristics of cirrhotic patients according to different sarcopenia statuses are shown in Supplementary Table S1. The BMI and HB of patients with sarcopenia was significantly lower than those without sarcopenia. Compared with those without sarcopenia, patients with sarcopenia have a higher prevalence of ascites (54.7% vs. 70.6%, *p* = 0.005). The overall mortality of those with sarcopenia was higher than those without sarcopenia (19.3% vs. 38.9%, *p* < 0.001). The overall survival time of patients without sarcopenia was significantly better than those with sarcopenia (32.11 ± 15.26 months vs. 27.40 ± 14.11 months, *p* = 0.007). Survival analysis using the Kaplan–Meier method (Supplementary Figure S1) also showed that the overall survival rates were significantly better in the non-sarcopenia group than in the sarcopenia group (Log-rank *p* < 0.001). After multivariate Cox analysis, sarcopenia (HR 1.950, 95%CI 1.166–3.261, *p* = 0.011) was an independent risk factor for the prognosis of patients with liver cirrhosis (Supplementary Table S2).

### Changes in sarcopenia for patients with cirrhosis

At the second assessment, 10.10% (31/307) patients without sarcopenia developed sarcopenia, 27.69% (85/307) with persistent sarcopenia status, while 13.03% (40/307) patients with sarcopenia developed non-sarcopenia, and 49.19% (151/307) with persistent non-sarcopenia status. Table 1 shows the demographic, nutritional, and clinical characteristics of all participants according to changes in sarcopenia at the second assessment. At the second CT assessment, the patients with new-onset sarcopenia and persistent sarcopenia had a significantly lower BMI than those without sarcopenia (*p* < 0.001). Although the MELD scores were no difference between different statuses of sarcopenia, the Child-Pugh scores for patients with persistent sarcopenia and new-onset sarcopenia were significantly higher than those without sarcopenia at the second assessment. In addition, patients with persistent sarcopenia and new-onset sarcopenia have a higher prevalence of HE (*p* = 0.003), ascites (*p* = 0.006), and SBP (*p* = 0.008) than those without sarcopenia at the second assessment. The level of ALB (*p* = 0.011) and HB (*p* < 0.001) also have differences in different sarcopenia statuses at the second assessment.

### Longitudinal changes in clinical characteristics

Table 1 also shows the clinical characteristics of all patients at the initial and second evaluation. For patients with persistent non-sarcopenia, the MELD score (13.64 ± 5.52 vs. 15.59 ± 5.95, *p* = 0.004) and Child-Pugh score (7.76 ± 2.20 vs. 8.69 ± 2.02, *p* < 0.001) were all significantly lower at the second evaluation compared the baseline. For sarcopenia to non-sarcopenia, the MELD score (13.99 ± 5.82 vs. 16.68 ± 5.98, *p* = 0.045) and Child-Pugh score (7.73 ± 2.16 vs. 9.05 ± 2.12, *p* = 0.007) were also significantly lower at the second evaluation compared the initial scan. However, the MELD score and Child-Pugh score were no different between the initial and second evaluations in patients with persistent sarcopenia and new-onset sarcopenia. The prevalence of SBP at the second evaluation was lower than initial evaluation for patients with persistent non-sarcopenia (23.3% vs. 41.1%, *p* = 0.001) and sarcopenia to non-sarcopenia (12.5% vs. 37.5%, *p* = 0.010). The prevalence of HE was significantly higher at the second evaluation than first evaluation for patients with persistent sarcopenia (43.0% vs. 15.1%, *p* = 0.001). In patients with persistent non-sarcopenia, the level of ALB (*p* < 0.001), PA (*p* = 0.044), and HB (*p* = 0.041) were also significantly higher at the second assessment than at baseline. The level of ALB in patients from sarcopenia to non-sarcopenia was also higher at the second assessment than the initial assessment (*p* = 0.024).

### Associations between changes in sarcopenia and prognosis

**Figure 2:**
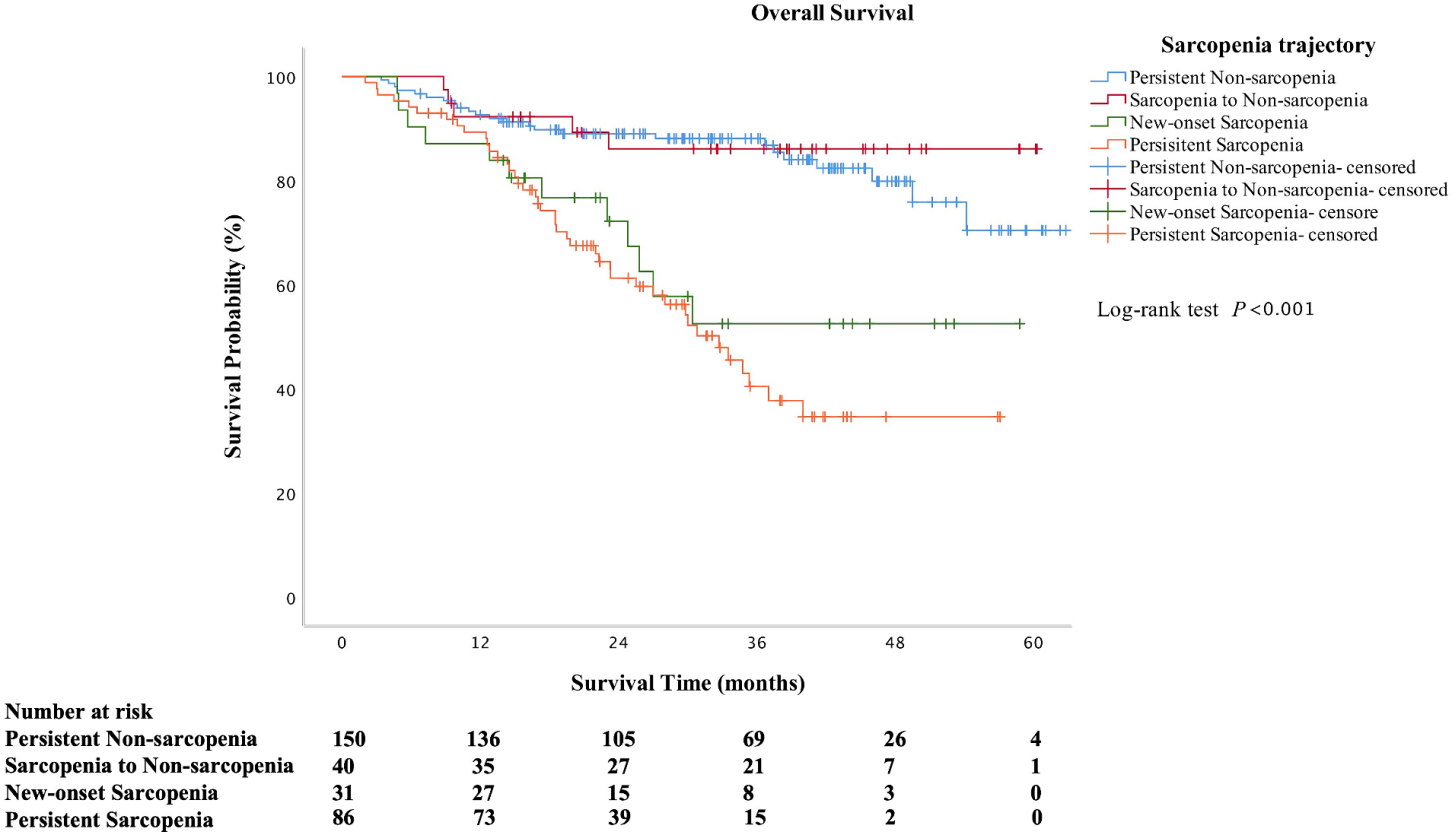
Kaplan–Meier curves of overall survival according to the change in sarcopenia status (log-rank test: *p* < 0.001 for overall survival).

A total of 84 (27.4%) patients died during the follow-up. The overall mortality in persistent sarcopenia and new-onset sarcopenia was significantly higher than in persistent non-sarcopenia and sarcopenia to non-sarcopenia (*p* < 0.001). The overall survival time was also lower in persistent sarcopenia and new-onset sarcopenia than in those without sarcopenia at the second assessment (*p* < 0.001). As shown in Figure 2, the overall survival rate was significantly lower in the persistent sarcopenia and new-onset sarcopenia than in the non-sarcopenia group and sarcopenia to non-sarcopenia group (log-rank test, *p* < 0.001). After multivariate Cox analysis, persistent sarcopenia (HR 5.799, 95%CI 1.563–21.521, *p* = 0.009) and new-onset sarcopenia (HR 5.205, 95%CI 1.482–18.282, *p* = 0.010) were identified as poor prognostic factors for cirrhotic patients (Table 2).

**Table 2:** Cox regression analyses of risk factors associated with overall mortality in cirrhosis.

	Univariate			Multivariate		
	HR	95%CI	*p*-value	HR	95%CI	*p*-value
Gender male	0.684	0.443–1.055	0.086			
Age	1.012	0.993–1.031	0.213			
BMI	0.927	0.876–0.980	0.008	1.065	0.998–1.137	0.059
Sarcopenia trajectory						
Non-Sarcopenia	Reference			Reference		
Sarcopenia	4.253	2.559–7.070	<0.001	5.799	1.563–21.521	0.009
Sarcopenia to non-sarcopenia	0.761	0.290–1.995	0.579	1.619	0.259–10.131	0.607
Non-sarcopenia to Sarcopenia	3.023	1.509–6.055	0.002	5.205	1.482–18.282	0.010
MELD score	1.095	1.064–1.127	<0.001	0.927	0.819–1.050	0.232
Child-Pugh score	1.386	1.262–1.521	<0.001	1.805	1.141–2.855	0.012
HE	2.384	1.548–3.671	<0.001	0.724	0.200–2.622	0.623
Ascites	3.268	1.915–5.577	<0.001	1.102	0.233–5.202	0.903
SBP	3.653	2.371–5.629	<0.001	2.550	0.619–10.502	0.195
AST	1.000	0.998–1.003	0.776			
ALT	0.999	0.983–1.005	0.706			
ALB	0.939	0.907–0.971	<0.001	1.104	0.997–1.223	0.058
TBIL	1.006	1.004–1.008	<0.001	1.001	0.993–1.010	0.729
PA	0.987	0.981–0.994	<0.001	0.995	0.980–1.010	0.495
Cr	1.003	1.000–1.006	0.079			
NLR	1.046	1.001–1.093	0.047	0.995	0.896–1.105	0.929
HB	0.979	0.971–0.987	<0.001	0.981	0.959–1.003	0.085

MELD, model for end-stage liver disease; BMI, body mass index; AST, aspartate amino transferase; ALT, alanine aminotransferase; TBIL, total bilirubin; ALB, albumin; PA, prealbumin; Cr, creatinine; NLR, the neutrophil-to-lymphocyte ratio; HB, hemoglobin; SBP, spontaneous peritonitis; HE, Hepatic Encephalopathy; HR, hazard ratios; CI, confidence interval.

### Independent predictors of new-onset sarcopenia

At the second evaluation, 17.13% (31/181) of patients with non-sarcopenia at initial assessment developed to new-onset sarcopenia. Using linear regression, variables that associated with the new-onset sarcopenia are shown in Table 3. Compared with persistent no-sarcopenia, the patients with new-onset sarcopenia had lower levels of BMI, L3-SMI, PA, Cr, and HB. The prevalence of HE was significantly higher than those with persistent no-sarcopenia. There were also significant differences in etiology between patients with persistent non-sarcopenia and those with new-onset sarcopenia (*p* = 0.004). Multivariate logistic regression analyses showed that the etiology of cirrhosis and the initial L3-SMI were independent risk factors for new-onset sarcopenia.

**Table 3:** Univariate and multivariate logistic regression analysis of new-onset sarcopenia in cirrhotic patients.

	Univariate			Multivariate	
	Persistent non-sarcopenia *N* = 150	New-onset sarcopenia *N* = 31	*p*-value	*OR* (95% CI)	*p*-value
Gender male, *n* (%)	102 (68.0)	18 (58.1)	0.287		
Age, years	56.63 ± 12.03	59.94 ± 11.85	0.165		
BMI, kg/m^2^	24.23 ± 4.82	21.09 ± 3.97	<0.001		
L3-SMI, cm^2^/m^2^	49.41 ± 6.86	44.20 ± 4.81	<0.001	0.857 (0.767–0.957)	0.006
MELD score	15.59 ± 5.95	15.65 ± 6.51	0.965		
Child-Pugh score	8.69 ± 2.02	8.77 ± 1.96	0.823		
Etiology					
Viral, *n* (%)	61 (40.7)	7 (22.6)	0.004	Reference	
Alcoholic, *n* (%)	39 (26.0)	8 (25.8)		2.971 (0.761–11.600)	0.117
PBC, *n* (%)	11 (7.3)	9 (29.0)		8.945 (1.899–42.138)	0.006
Others, *n* (%)	39 (26.0)	7 (22.6)		1.914 (0.494–7.423)	0.348
Ascites, *n* (%)	81 (54.0)	18 (58.1)	0.679		
HE, *n* (%)	29 (19.3)	11 (35.5)	0.049		
SBP, *n* (%)	61 (40.7)	13 (41.9)	0.896		
AST, U/L	36.00 (26.00, 67.00)	44.00 (27.00, 71.00)	0.706		
ALT, U/L	23.50 (15.00, 40.00)	19.00 (13.00, 36.50)	0.248		
TBIL, μmol/L	43.25 (25.13, 85.30)	46.90 (19.90, 140.40)	0.968		
ALB, g/L	30.35 (27.68, 33.83)	31.30 (28.70, 33.50)	0.245		
PA, mg/L	76.00 (60.00, 115.00)	63.00 (52.00, 75.00)	0.010		
Cr, μmol/L	62.00 (52.00, 74.25)	51.00 (46.00, 64.00)	0.024		
NLR	2.48 (1.59, 3.73)	2.82 (1.36, 4.02)	0.979		
HB, g/L	101.50 (79.00, 120.25)	88.00 (71.00, 109.00)	0.038		

OR, odds ratio; MELD, model for end-stage liver disease; BMI, body mass index; L3-SMI, third lumbar skeletal muscle index; AST, aspartate amino transferase; ALT, alanine aminotransferase; TBIL, total bilirubin; ALB, albumin; PA, prealbumin; Cr, creatinine; NLR, the neutrophil-to-lymphocyte ratio; HB, hemoglobin; SBP, spontaneous peritonitis; HE, Hepatic Encephalopathy; HR, hazard ratios; CI, confidence interval. PBC, primary biliary cholangitis.

## Discussion

Sarcopenia is a syndrome characterized by the progressive loss of skeletal muscle mass, strength, and function (29, 30). Sarcopenia status may constantly change; therefore, the longitudinal assessment of sarcopenia is important. In this research, we found that skeletal muscle mass is dynamically changing in patients with cirrhosis. Improvements in sarcopenia were accompanied by improvements in disease severity. Compared with those of persistent non-sarcopenia, both cirrhotic patients with new-onset sarcopenia and persistent sarcopenia were associated with a higher risk of mortality.

Although sarcopenia is important, there is no agreement on how to accurately diagnose sarcopenia in clinical settings (31). Although most working groups recommend considering both muscle mass and muscular function for the diagnosis of sarcopenia, almost all studies in cirrhotic patients have investigated sarcopenia using measures of muscle mass alone (17). Based on the available data on liver disease, some guidelines developed a consensus definition for the operationalization of sarcopenia in liver disease as the phenotypic manifestation of loss of muscle mass alone (4, 17, 26). Radiographic image analysis is considered the most accurate technique for quantifying muscle mass and defining sarcopenia (32). Compared with other evaluation tools, the L3-SMI has the advantages of being objective, quantifiable, accurate, and non-invasive, and is a good indicator of protein malnutrition and sarcopenia (19). CT scans have become common as a routine follow-up evaluation of patients with cirrhosis. Thus, L3-SMI has good accessibility and reproducibility for longitudinal assessment of sarcopenia. Therefore L3-SMI was used in the current study to diagnose sarcopenia and to evaluate the longitudinal changes in sarcopenia.

The prevalence of sarcopenia in cirrhotic patients is considerably reported with a wide variety due to the differences in sample size, ethnicity, diagnostic criteria, assessment procedures, and diagnostic thresholds for sarcopenia (26, 30, 33, 34). A previous meta-analysis found the prevalence in patients with cirrhosis was 37.5% (35). One research included 480 Chinese cirrhotic patients and reported 22.5% had sarcopenia diagnosed by the cut-off values of L3-SMI based on general Chinese adults (19). Another study performed on 115 Indian patients undergoing liver transplantation found the prevalence of sarcopenia was 47.8% (36). The prevalence of sarcopenia at baseline for cirrhotic patients was 41.04%, which was comparable to that reported in previous studies (37–39). However, there is very limited data assessing the temporal changes of muscle area in cirrhosis (17, 23). This study demonstrated that 17.12% developed sarcopenia among cirrhotic patients without sarcopenia at baseline after the second assessment. In addition, 31.75% of patients with initial sarcopenia converted to non-sarcopenia after the second assessment. Another study found that new-onset sarcopenia is seen in up to 25% of the patients after liver transplantation (40). Sarcopenia status can constantly change, and the longitudinal assessment of sarcopenia is important. Persistent muscle loss may indicate a failure of adaptive mechanisms to decelerate the rate of muscle loss and the lack of any interventions to reverse muscle loss (23).

In patients with liver cirrhosis, sarcopenia was associated with a high risk of cirrhosis-related complications, a greater length of stay and hospital costs, and higher mortality (18, 41–44). A meta-analysis showed sarcopenia was associated with an increased risk of mortality (35). Furthermore, the presence of sarcopenia is an independent predictor of multiple adverse clinical outcomes in cirrhotic patients (19–22). In cross-sectional studies for cirrhotic patients, muscle area has been correlated to survival (23, 45). Similarly, patients with sarcopenia also had a higher prevalence of mortality than those without sarcopenia in this study. Sarcopenia status may constantly change; therefore, these prognostic values for mortality might also change over time (30). From the Kaplan–Meier curves, the overall survival rate was significantly lower in the persistent sarcopenia and new-onset sarcopenia than in the non-sarcopenia group and sarcopenia to non-sarcopenia group in our study. The survival curve of sarcopenia resolved was above in persistent non-sarcopenia after the follow-up of 40 months. However, the survival time (33.19 ± 15.04 months vs. 34.64 ± 15.18 months, *p* = 0.720) and the overall mortality (15.3% vs. 15.0%, *p* = 0.894) in those two groups were no difference. We speculate that the large difference in the number of patients between the two groups may have led to some bias in the long-term follow-up. A previous study focused on patients undergoing hemodialysis found that longitudinal associations were observed between new-onset, persistent sarcopenia, and cognitive impairment (30). Another study showed that sarcopenia is progressive and that sarcopenia from longitudinal measures are predictor of clinical outcomes (23). Although, the previous study mainly explored the relationship between the etiology of liver disease and the rate of muscle loss, further study on the relationship between the longitudinal changes in sarcopenia and prognosis did not conduct (23). In addition, our finding showed that persistent sarcopenia and new-onset sarcopenia were associated with an approximately 5-fold higher risk of death in patients with cirrhosis. These results showed that in addition to sarcopenia at a certain time in point, changes in sarcopenia status could be also an important basis for predicting clinical outcomes, which is a novel finding that extends the meanings of previous studies (30).

The evaluation of the natural progression and predictors of accelerated decline were important parts for longitudinal assessment of sarcopenia (17). Previous studies have been reported that sarcopenia is associated with the severity of liver disease (19, 46). The association between changes in liver disease severity and changes in skeletal muscle mass is unclear (23). For patients with persistent non-sarcopenia and sarcopenia to non-sarcopenia, the MELD score and Child-Pugh score at the second assessment were all significantly lower than the initial assessment. In liver disease, the levels of albumin and pre-albumin generally reflect the severity of the liver disease (47). In patients with persistent non-sarcopenia and sarcopenia to non-sarcopenia, the level of albumin is significantly higher at the second assessment than at baseline. In the second assessment, the prevalence of SBP in persistent non-sarcopenia and sarcopenia to non-sarcopenia are significantly lower than the initial assessment, and the prevalence of HE in persistent sarcopenia is significantly higher than the baseline. Thus, improvements in sarcopenia are accompanied by improvements in disease severity. The increase or decrease in the incidence of complications of cirrhosis may also be related to the changes of sarcopenia. But more research is needed to prove this argument in the future. We also noted that patients with cirrhosis who had lower muscle mass at the initial scan were more likely to develop new-onset sarcopenia. This suggests that some patients, although not diagnosed with sarcopenia at initial evaluation, are at higher risk of developing new-onset sarcopenia due to their low initial skeletal muscle mass. Single assessment at a point is likely to miss these patients, and longitudinal assessment could help clinicians to identify patients with high-risk factors and take more active intervention and follow-up. Cholestasis may result impaired metabolism and malabsorption of long-chain fatty acids and fat-soluble vitamin deficiency (17, 48). The cholestatic liver disease led to elevated serum bile acid levels that may induce skeletal muscle atrophy through the bile acid receptor G protein–coupled bile acid receptor 1 (26, 49). Similarly, patients with PBC are more likely to have new-onset sarcopenia than patients with other liver diseases. Therefore, more attention needs to be given to these patients.

However, there are several limitations to this study. First, the retrospective nature of the study is an inherent limitation. Even considering sarcopenia status changes, the causal relationship could not be confirmed in an observational design. Second, although muscle function is an important part of sarcopenia, muscle function was not evaluated in this current study. Longitudinal assessment of muscle function and frailty should be evaluated in further study. Third, the longitudinal assessment for sarcopenia depended only on two points in time, and any treatments in cirrhotic patients were not evaluated in this retrospective study. Long-term longitudinal evaluation of sarcopenia is needed to evaluate the natural progression or response to treatment.

In conclusion, sarcopenia was common in cirrhotic patients and was associated with poor clinical outcomes. Sarcopenia is a dynamically changing process in patients with cirrhosis. Persistent sarcopenia and new-onset sarcopenia were independently and robustly associated with mortality in short term.

## Data availability statement

The raw data supporting the conclusions of this article will be made available by the authors, without undue reservation.

## Ethics statement

The studies involving humans were approved by Ethics Committee (seal) of Beijing Youan Hospital, Capital Medical University. The studies were conducted in accordance with the local legislation and institutional requirements. Written informed consent for participation was not required from the participants or the participants’ legal guardians/next of kin in accordance with the national legislation and institutional requirements.

## Author contributions

MJ: Data curation, Formal analysis, Software, Writing – original draft. XH: Data curation, Formal analysis, Writing – original draft, Writing – review & editing. MW: Data curation, Writing – original draft. JW: Data curation, Writing – review & editing. XX: Data curation, Writing – review & editing. JL: Validation, Writing – review & editing. QM: Formal analysis, Writing – review & editing.
