# Supplementary material for: Longitudinal changes in sarcopenia was associated with survival among cirrhotic patients
Source: Front Nutr. 2024 May 30;11:1375994. doi: 10.3389/fnut.2024.1375994 (PMC11169581; doi:10.3389/fnut.2024.1375994)
Supplement: Supplementary file 1 [file Data_Sheet_1.docx]

**Table S1: Clinical characteristics of cirrhotic patients according to different sarcopenia status.**

|  | **Overall patients**  **N=307** | **Normal**  **N=181** | **Sarcopenia**  **N=126** | ***P*-value** |
| --- | --- | --- | --- | --- |
| Gender male, n (%) | 188( 61.2) | 120 (66.3) | 68 (54.0) | 0.029 |
| Age, years | 57.35±11.81 | 57.20±12.03 | 57.56±11.52 | 0.795 |
| BMI, kg/m^2^ | 22.09±4.74 | 23.69±4.82 | 19.78±3.53 | <0.001 |
| L3-SMI(male), cm^2^/m^2^ | 45.84±8.62 | 50.57±6.59 | 37.49±4.42 | <0.001 |
| L3-SMI(female), cm^2^/m^2^ | 38.96±7.51 | 44.47±5.37 | 33.18±4.47 | <0.001 |
| MELD score | 15.57±6.10 | 15.63±6.04 | 15.49±6.21 | 0.848 |
| Child-Pugh score | 8.69±2.03 | 8.70±2.01 | 8.68±2.07 | 0.935 |
| **Etiology** |  |  |  |  |
| Viral, n (%) | 107(34.9) | 68(37.6) | 39(31.0) | 0.132 |
| Alcoholic, n (%) | 88(28.7) | 47(26.0) | 41(32.5) |  |
| PBC, n (%) | 42(13.7) | 20(11.0) | 22(17.5) |  |
| others, n (%) | 70(22.8) | 46(25.4) | 24(19.0) |  |
| **Clinical outcomes** |  |  |  |  |
| HE, n (%) | 64 (20.8) | 40 (22.1) | 24 (19.0) | 0.517 |
| Ascites, n (%) | 188 (61.2) | 99 (54.7) | 89 (70.6) | 0.005 |
| SBP, n (%) | 122 (39.7) | 74 (40.9) | 48 (38.1) | 0.623 |
| Death, n (%) | 84 (27.4) | 35 (19.3) | 49 (38.9) | <0.001 |
| OS, months | 30.18±14.96 | 32.11±15.26 | 27.40±14.11 | 0.007 |
| **Serological examination** |  |  |  |  |
| AST, U/L | 35.00(25.00,61.80) | 37.00 (26.00, 67.00) | 32.50 (24.00, 51.50) | 0.056 |
| ALT, U/L | 20.00(14.00,35.00) | 23.00 (15.00, 38.50) | 18.00 (13.00, 29.00) | 0.005 |
| TBIL, μmol/L | 42.90(23.60, 84.30) | 43.30 (23.90, 86.30) | 37.55 (22.73, 77.10) | 0.370 |
| ALB, g/L | 30.70 (27.90, 34.40) | 30.60 (28.00, 33.80) | 30.95 (27.88, 34.88) | 0.508 |
| PA, mg/L | 76.00 (58.00, 109.25) | 73.00 (58.00, 112.00) | 81.00 (57.50,105.50) | 0.805 |
| Cr, μmol/L | 60.00 (49.00, 74.00) | 60.00 (50.00,74.00) | 59.00 (48.00, 72.50) | 0.638 |
| NLR | 2.49 (1.59, 4.29) | 2.49 (1.58, 3.75) | 2.55 (1.63, 5.05) | 0.414 |
| HB, g/L | 91.00 (72.00, 113.00) | 97.00 (78.00, 118.00) | 85.00 (69.00, 107.25) | 0.001 |

**Table S2: Cox regression analyses of risk factors associated with overall mortality in cirrhosis at baseline.**

|  | **Univariate** | | |  | **Multivariate** | | |
| --- | --- | --- | --- | --- | --- | --- | --- |
|  | **HR** | **95%CI** | ***P*-value** |  | **HR** | **95%CI** | ***P*-value** |
| Gender male | 0.684 | 0.443-1.055 | 0.086 |  |  |  |  |
| Age, years | 1.012 | 0.993-1.031 | 0.213 |  |  |  |  |
| BMI | 0.974 | 0.924-1.027 | 0.331 |  |  |  |  |
| Sarcopenia | 2.296 | 1.483-3.556 | <0.001 |  | 1.961 | 1.174-3.277 | 0.010 |
| MELD score | 1.057 | 1.023-1.093 | 0.001 |  | 1.012 | 0.948-1.081 | 0.716 |
| Child-Pugh score | 1.172 | 1.057-1.300 | 0.003 |  | 1.025 | 0.818-1.286 | 0.828 |
| HE | 0.956 | 0.537-1.700 | 0.877 |  |  |  |  |
| Ascites | 1.295 | 0.814-2.059 | 0.276 |  |  |  |  |
| SBP | 1.780 | 1.157-2.739 | 0.009 |  | 1.632 | 0.981-2.716 | 0.059 |
| AST | 0.999 | 0.995-1.003 | 0.614 |  |  |  |  |
| ALT | 0.995 | 0.988-1.001 | 0.122 |  |  |  |  |
| TBIL | 1.001 | 1.000-1.003 | 0.063 |  |  |  |  |
| ALB | 0.957 | 0.916-0.999 | 0.045 |  | 1.019 | 0.949-1.093 | 0.607 |
| PA | 0.990 | 0.984-0.997 | 0.005 |  | 0.993 | 0.984-1.002 | 0.115 |
| Cr | 1.000 | 0.998-1.003 | 0.742 |  |  |  |  |
| NLR | 1.002 | 1.000-1.003 | 0.040 |  | 1.001 | 0.999-1.002 | 0.372 |
| HB | 0.990 | 0.982-0.998 | 0.017 |  | 0.994 | 0.984-1.005 | 0.308 |

**Figure S1:** **Kaplan–Meier curves of overall survival according to the sarcopenia (log-rank test: *P* <0.001 for overall survival).**
